# Trends in descriptions of palliative care in the cancer clinical practice guidelines before and after enactment of the Cancer Control Act (2007): content analysis

**DOI:** 10.1186/s12904-019-0391-z

**Published:** 2019-01-12

**Authors:** Miwa Hinata, Kikuko Miyazaki, Natsuko Kanazawa, Kumiko Kito, Sachiko Kiyoto, Manako Konda, Akira Kuriyama, Hiroko Mori, Sachiko Nakaoka, Akiko Okumura, Hironobu Tokumasu, Takeo Nakayama

**Affiliations:** 1 Department of Hospital Pharmaceutics, Showa University, Tokyo, Japan; 2 Department of Health Informatics, Kyoto University School of Public Health, Yoshida honcho sakyo-ku, Kyoto, Japan; 3 Clinical Research Center, National Hospital Organization, Tokyo, Japan; 4 Department of Food and Life science, Azabu University, Kanagawa, Japan; 5 Department of Breast Oncology, National Hospital Organization Shikoku Cancer Center, Ehime, Japan; 6 Department of Preventive Services, Kyoto University School of Public Health, Kyoto, Japan; 7 Tokyo Metropolitan Institute of Gerontology Human care research Team, Tokyo, Japan; 8 Department of EBM and Guidelines, Japan Council for Quality Health Care, Tokyo, Japan; 9 Department of Consultation, Kurashiki Clinical Research Institute, Okayama, Japan

**Keywords:** Palliative care, Cancer clinical practice guideline, Cancer control act [2007], Content analysis, Qualitative research

## Abstract

**Background:**

Palliative care was a priority issue in the Cancer Control Act enacted in 2007 in Japan, and this has resulted in efforts being made toward educational goals in clinical settings. An investigation of how descriptions of palliative care for the treatment of cancer have changed in clinical practice guidelines (CPGs) could be expected to provide a better understanding of palliative care-related decision-making. This study aimed to identify trends in descriptions of palliative care in cancer CPGs in Japan before and after enactment of the Cancer Control Act.

**Methods:**

Content analysis was used to count the lines in all relevant CPGs. We then compared the number of lines and the proportion of descriptions mentioning palliative care at two time points: the first survey (selection period: February to June 2007) and the second survey (selection period: February to December 2015). Descriptions from the CPGs were independently selected from the Toho University Medical Media Center and Medical Information Network Distribution Service databases, and subsequently reviewed, by two investigators.

**Results:**

Descriptions were analyzed for 10 types of cancer. The proportion of descriptions in the first survey (4.4%; 933/21,344 lines) was similar to that in the second survey (4.5%; 1325/29,269 lines).

**Conclusions:**

After the enactment of the Cancer Control Act, an increase was observed in the number, but not in the proportion, of palliative care descriptions in Japanese cancer CPGs. In the future, CPGs can be expected to play a major role in helping cancer patients to incorporate palliative care more smoothly.

## Background

According to the Institute of Medicine in the United States, clinical practice guidelines (CPGs) are statements that include recommendations intended to optimize patient care [1]. They are informed by a systematic review of evidence and an assessment of the benefits and harms of alternative care options. CPGs generally cover 60–95% of clinical conditions [2]. After a 1997 report on the assessment of health technology by a working group of the Ministry of Health, Labour and Welfare in Japan, [3] the development of CPGs began to increase in 1999 [4]. CPGs in many medical disciplines, including cancer, have now been developed, mainly by academic and professional associations, using evidence-based approaches [5, 6]. CPGs are periodically revised to include the most up-to-date information [7]. These CPGs help support clinical decision-making and advocate the role of continuing education for healthcare providers [8].

In 1981, cancer surpassed stroke as the leading cause of death in Japan [9]. Cancer is a major health concern among people in Japan. For healthcare systems, a quantitative infrastructure (e.g., cancer screening) has been under constant development since 1984; however, the qualitative infrastructure (e.g., patients and support from their families) remains insufficient [10]. Enacted in 2007, the Cancer Control Act [11] aims to promote comprehensive planning for cancer management based on cancer prevention, early cancer detection, furthering cancer research, and eliminating disparities in cancer treatment [11]. Cancer control programs emphasize palliative care as a priority issue and aim at “promoting palliative care from the time when cancer is diagnosed” [11, 12]. An investigation of how descriptions of palliative care have changed in cancer CPGs in Japan could provide a better understanding of palliative care-related decision-making in clinical practice [1, 13, 14].

A study reviewing 91 CPGs for nine life-threatening diseases (including breast, colorectal, prostate, and lung cancers) published between 1987 and 2002 in the United States reported that palliative and end-of-life care were seldom mentioned [15]. However, no similar studies have previously been reported in Japan. Therefore, the aim of the present study was to identify trends in descriptions about palliative care in Japanese cancer CPGs before and after enactment of the Cancer Control Act in 2007.

## Methods

### Design

In the present study, we used content analysis of existing literature [16, 17] to compare the number of lines and proportion of descriptions mentioning palliative CPGs before and after enactment of the Cancer Control Act in 2007.

### The first survey (selection period: February to June 2007)

Cancer CPGs published between January 2002 and December 2006 were analyzed. Databases from the Toho University Medical Media Center [18] (search date: January 18, 2007) and the Medical Information Network Distribution Service (MINDS) [19, 20] were reviewed. Assuming that the cancer CPGs were tools to provide information sharing for decision-making by healthcare providers, patients, and their families, cancer CPGs that were available not only to healthcare providers, but also to the general public, were selected. The following cancer CPGs were excluded from analysis: 1) those from foreign countries that were translated into Japanese, 2) those developed for the purpose of using specific cancer treatment regimens, for example, “for optimal use of a specific anticancer drug” or “for appropriate use of thalidomide in multiple myeloma”, 3) those not readily available to the general public, for example, “Cancer CPGs published in academic or professional journals”, and 4) guideline manuals. Palliative care was defined based on previous reports, [13] namely, as “care given to a patient when there is no response to curative treatment and life-expectancy is less than one year.” We defined 17 criteria for selecting palliative care descriptions from cancer CPGs (Table 1).

**Table 1:** Domains of palliative care

1.	Non-pain symptom assessment and management (dyspnea, nausea and vomiting, delirium, fatigue, etc.)
2.	Pain assessment and management
3.	Natural history (prognosis, time course, mode of death, and symptoms)
4.^a^	Palliative care
5.^a^	Palliative operation/symptomatic treatment
6.	Necrology (death statistics, including gender, age at death, any racial disparities)
7.	Social issues (interpersonal relationships with spouses or partners, family, and friends supporting these relationships)
8.	Care setting (option for location of end-of-life care, referral to hospice, funeral arrangements)
9.	Psychological issues (depression, anxiety, fear, loneliness, emotional awareness)
10.	Financial issues (cost to patient and family, not insurer or societal cost)
11.	Patient or family values (any discussion regarding patient and/or family goals and values, including advanced directives and “do not resuscitate” orders)
12.	Goal of care (goals of care related to quality of life and end-of-life care)
13.	Physician communication with patient/family (including communication with patient and family about personal grief and bereavement)
14.	Ethics, laws, and policies (individual vs. organization ethics, patients’ self-determination, double effect, legal aspects of withdrawal, and withholding of life support)
15.	Physician roles in advocacy and policy (including pronouncement, autopsy, organ donation, advocacy, and changing institutional policy)
16.	Spiritual issue (abandonment, completion of tasks, acceptance, religious tasks, and choices)
17.	Family roles and responsibilities (communication of patient and family member roles during the process, grief and bereavement, caregiver roles and support)

^a^We included the following two criteria: “Palliative care” and “Palliative operation/symptomatic treatment”, which could not be classified by the 15 criteria; problems related to the boundary area are also included

Fifteen criteria were used based on those reported by Mast et al [13]. In addition, we included the following two criteria: “Palliative care” and “Palliative operation/symptomatic treatment”, which could not be classified by the 15 criteria; we also included problems related to the boundary area. Selection was based on whether the descriptive content met the definitions and selection criteria. Even if “palliative care” was not directly mentioned, if the description corresponded to the selection criteria, it was selected for analysis. The amount of text was quantified as follows: 1) a single word matching one of 17 criteria was counted as one; 2) the total number of lines of relevant descriptions describing one of the 17 criteria was counted; and 3) the context of references to the cancer pain guidelines by the Japanese Society for Palliative Medicine, [21, 22] as well as a description of participation of the society on “guideline executive committee” and “external review committee” and “guideline development committee”; 4) descriptions of “palliative care from the time when cancer is diagnosed” after enforcement of the Cancer Control Act; and 5) presence of the following four terms: palliative care, best supportive care (BSC), palliative therapy, and symptomatic treatment.

The proportion of descriptions was calculated by dividing by the total number of lines for each criterion. Since Japanese cancer CPGs are in the same form, we adopted this method to compare quantitatively the number of descriptions of palliative care. We defined two classifications: “minimal content”, which referred to words that did not include any specific information regarding the 17 criteria, and “helpful content”, which referred to a description that included specific and useful information about the 17 criteria. If classification between “minimal content” and “helpful content” was difficult, we classified the description as “helpful content”. Descriptions were independently selected from each CPG by two investigators.

These data were then compared by the two investigators, and any disagreement was resolved through discussion before the data analysis. If any discrepancies between the two investigators remained, a consensus was reached through deliberation with all study members.

### The second survey (selection period: February to December 2015)

We evaluated the most current versions of the CPGs at the time of review in 2015 (search date: March 4, 2015). The data were analyzed in the same manner as that in the first survey.

## Results

### Selected clinical practice guidelines (CPGs)

**Fig. 1:**
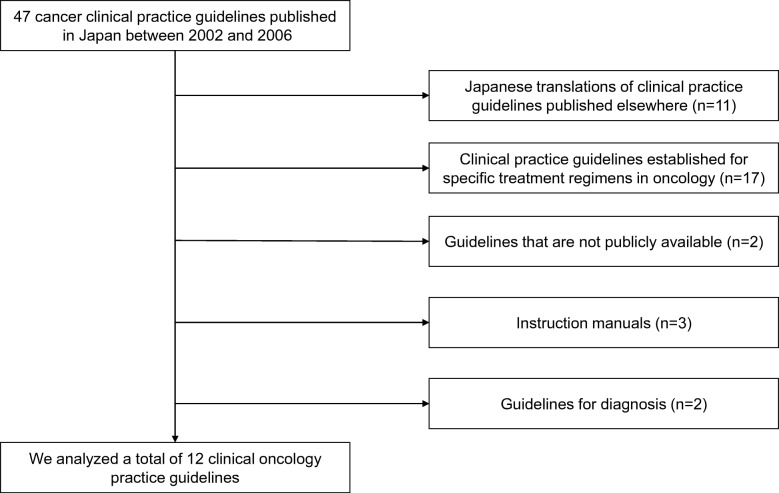
Selection of Clinical Practice Guidelines

Among 47 cancer CPGs in the first survey, 35 were excluded based on the exclusion criteria; therefore, we finally analyzed 12 CPGs for the following 10 types of cancer: esophageal, gastric, breast (chemotherapy, surgery, and radiation therapy), colorectal, lung, liver, prostate, pancreatic, uterine, and ovarian (Fig. 1). In the second survey, CPGs for the same types of cancer included in the first survey were examined (Table 2). However, the editing formats for the breast cancer CPGs differed between the first and second surveys. Among all five separate volumes of breast cancer CPGs (chemotherapy, radiation therapy, surgery, epidemiology/diagnosis, and prevention) in the first survey, we analyzed three volumes (chemotherapy, radiation therapy, and surgery). For the second survey, we analyzed the treatment volume among two volumes (the other was for diagnosis). All CPGs were developed by relevant clinical societies.

**Table 2:** Editor and publication year of the clinical practice guidelines (CPGs) (Japanese)

Guideline	2007 survey (Edition)	2015 survey (Edition)	Editor
Guidelines for Diagnosis and Treatment of Carcinoma of the Esophagus	2002 (1st)	2012 (3rd)	The Japan Esophageal Society
Gastric Cancer treatment guidelines	2004 (2nd)	2014 (4th)	Japanese Gastric Cancer Association
Guideline for Ovarian Cancer Treatment	2004 (1st)	2010 (3rd)	The Japan Society of Gynecologic Oncology
The Japanese Breast Cancer Society Clinical Practice Guideline^a^	Chemo 2004 (1st)	2013 (Chemo 4th) (Radiation 3rd) (Surgery 3rd)	The Japanese Breast Cancer Society
	Radiation 2005 (1st)		
	Surgery 2005 (1st)		
JSCCR Guidelines for the Treatment of Colorectal Cancer	2005 (1st)	2014 (3rd)	The Japanese Society for Cancer of the Colon and Rectum
Guideline for Diagnosis and Treatment of Lung Cancer	2005 (2nd)	2014 (3rd)	The Japan Lung Cancer Society
Clinical Practice Guidelines for Hepatocellular Carcinoma	2005 (1st)	2013 (3rd)	The Japan Society of Hepatology
Evidence-based Clinical Practice Guidelines for Prostate Cancer	2006 (1st)	2012 (2nd)	The Japanese Urological Association
EBM-based Clinical Guidelines for Pancreatic Cancer	2006 (1st)	2013 (3rd)	Japan Pancreas Society
Evidence-based Guidelines for Treatment of Uterine Body Neoplasm	2006 (1st)	2013 (3rd)	The Japan Society of Gynecologic Oncology

^a^Among all five breast cancer CPGs (chemotherapy, radiation therapy, surgery, epidemiology/diagnosis, and prevention), we analyzed three volumes (chemotherapy, radiation therapy, and surgery). The treatment sections from two CPGs (treatment, diagnosis) included similar content between the first and second surveys

### Proportion of palliative care descriptions

We found 21,344 lines and 29,269 lines from all examined CPGs for first and second surveys, respectively. The total number of lines about palliative care in all guidelines increased from 933 in the first survey to 1325 in the second. However, the proportion of descriptions in the first survey (4.4%) was very similar to that in the second (4.5%) (Table 3). The number of “minimal content” descriptions increased 1.85-fold (from 75 to 139 lines) and the proportion of descriptions increased 1.34-fold (from 0.35 to 0.47%). The number of “helpful content” descriptions increased 1.38-fold (from 858 to 1186 lines) and the proportion of descriptions increased 1.01-fold (from 4.01 to 4.05%).

**Table 3:** Comparison of the descriptions of palliative care in the clinical practice guidelines of 2007 and 2015

Clinical practice guideline	Survey year	Minimal content (lines)	Helpful content (lines)	Total volume of guideline (lines)	Palliative care description^a^ (%)
Prostate cancer	2007	24	253	4272	6.5
	2015	15	312	3646	9.0
Lung cancer	2007	15	128	3866	3.7
	2015	9	98	3101	3.5
Pancreatic cancer	2007	4	148	1010	15.0
	2015	9	222	2552	9.1
Gastric cancer	2007	2	64	461	14.3
	2015	4	46	892	5.6
Colorectal cancer	2007	15	70	758	11.2
	2015	20	126	1551	9.4
Uterine neoplasms	2007	2	35	1521	2.4
	2015	15	26	2749	1.5
Breast cancer	2007	8	91	3191	3.1
	2015	21	110	7648	1.7
Esophageal cancer	2007	0	56	599	9.3
	2015	30	138	1802	9.3
Ovarian cancer	2007	4	13	806	2.1
	2015	8	99	2113	5.1
Hepatocellular carcinoma	2007	1	0	4860	0.0
	2015	8	9	3215	0.5
Total	2007	75	858	21,344	4.4
	2015	139	1186	29,269	4.5

^a^we calculated palliative care descriptions (%) by adding minimal content lines and helpful content lines divided by the total volume of lines

### Comparison of selected clinical practice guidelines (CPGs)

Table 3 shows the number and proportion of descriptions by CPGs. Regarding the proportion of descriptions, that for ovarian cancer increased from 2.1% (17/806 lines) to 5.1% (107/2113 lines), and that for prostate cancer from 6.5% (277/4272 lines) to 9.0% (327/3646 lines). Conversely, that for pancreatic cancer decreased from 15.0% (152/1010 lines) to 9.1% (231/2552 lines), and that for gastric cancer from 14.3% (66/461 lines) to 5.6% (50/892 lines) (Table 3). Among all 12 CPGs in the first survey, the cancer pain guidelines from the Japanese Society for Palliative Medicine [21, 22] were only referred to in the prostate cancer CPG. However, in the second survey, this was referred to in five of the 10 CPGs (the prostate cancer, pancreatic cancer, colorectal cancer, gastric cancer, and esophageal cancer CPGs). In addition, the description of “palliative care from the time when cancer is diagnosed” stated in the Cancer Control Act was stipulated only in the colon CPG before implementation, compared with the esophageal cancer, stomach cancer, pancreatic cancer, and colon cancer CPGs after implementation. Furthermore, in the second survey, the number of guidelines describing the terms “palliative care”, “best supportive care (BSC)”, and “symptomatic treatment” had increased (Table 4).

**Table 4:** Comparison of terms related to palliative care in the clinical practice guidelines

	2007 survey (guidelines)	2015 survey (guidelines)
Palliative care	8	9
Best supportive care (BSC)	3	7
Palliative therapy	8	7
Symptomatic treatment	4	5

We confirmed the CPGs for the two sessions in 2007 and 2015 that were covered for the three points of the society (guideline executive committee, external review committee, and guideline development committee) regarding the relationship with the Palliative Medical Society. As a result, we confirmed that there was no mention of these items in CPGs at either time point. In addition, there was no description as to whether the guideline creator was a member of the Japanese Society for Palliative Medicine.

### Examination of descriptions

**Fig. 2:**
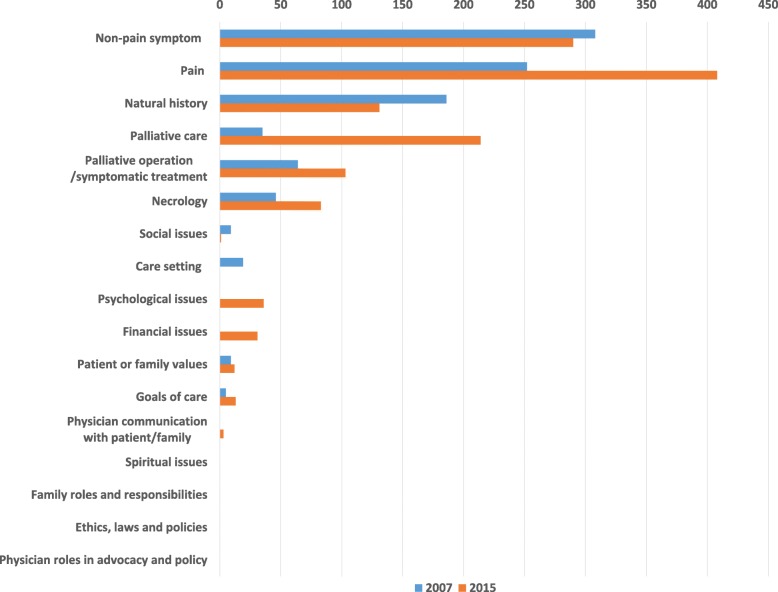
Number of Descriptions in Each Category for Palliative Care That Were Found in Clinical Practice Guidelines

The most commonly mentioned items were “pain” and “non-pain symptoms”, whereas four of the 17 items—“spiritual issues”, “family roles and responsibilities”, “ethics, laws, and policies”, and “physician roles in advocacy and policy”—were never mentioned (Fig. 2). “Helpful content” about “palliative care”, which provided information about best supportive care, controlled studies, and palliative chemotherapy, was mentioned much more frequently in the second than in the first survey.

## Discussion

Based on a comparison of cancer CPGs during two periods between 2007 and 2015, the number of palliative care descriptions increased as follows: 1.85-fold (from 75 to 139 lines) for “minimal content” and 1.38-fold (from 858 to 1186 lines) for “helpful content”. In addition, since the implementation of the Cancer Control Act in 2007, the number of CPGs describing “palliative care from the time when cancer is diagnosed” had increased; thus, recognition of the concept has come a long way.

This development was probably influenced by the significant changes in healthcare policies regarding palliative care over the last 10 years in Japan. One of these changes was the Cancer Control Act, [11] which was enacted in 2007 to improve cancer management and eliminate disparities in cancer treatment. With the enactment of the Cancer Control Act, palliative care teams were established at cancer hospitals in each region as part of a system to provide more appropriate palliative care. In addition, to enable all physicians who care for cancer patients to learn the basic principles of palliative care, palliative care workshops as part of the “Palliative care Emphasis program on symptom management and Assessment for Continuous medical Education (PEACE) project” have been conducted throughout Japan [23]. Moreover, a website has been developed, primarily by the cancer information center at the National Cancer Center, to provide information to patients [24]. These changes in social attitudes were seen after the promulgation of the Cancer Control Act, and these changes could be one of the factors that promoted improvement in palliative care.

However, the proportion of palliative care descriptions remained almost the same (4.4 and 4.5%). The reasons for this lack of substantial change in the proportion of descriptions are thought to be as follows: 1) there was a substantial increase in the total number of lines in CPGs, and 2) several CPGs encouraged reference to cancer pain guidelines (e.g., prostate cancer, pancreatic cancer, gastric cancer) [22]. In 2007, palliative care guidelines only described cancer pain treatment [21] in a single book. However, as of 2015, the following five CPGs were being widely used: drug therapy for cancer pain, [22] palliative sedation therapy, [25] gastrointestinal symptoms, [26] respiratory symptoms, [27] and infusion therapy [28].

From these circumstances, palliative care appears to be gaining more widespread attention. However, CPGs for palliative care are still developed independent of other cancer CPGs; no CPGs have been created by any cancer clinical society in collaboration with the Japanese Society for Palliative Medicine.

### Differences in cancer types

The number and proportion of palliative care descriptions varied widely among the CPGs depending on the type of cancer (Table 3). For example, from 2007, the CPGs for prostate cancer, the progression of which is usually slow, continuously had the highest number of palliative care descriptions among the 10 types of cancer [24, 29]. Moreover, many of the descriptions in the prostate cancer CPGs have focused on “non-pain symptoms.” As symptoms in prostate cancer progress, sexual function and urination are affected, bone metastases can occur, and quality of life (QOL) diminishes. Therefore, much has been written about palliative care to alleviate these symptoms.

On the other hand, the CPGs for hepatocellular carcinoma (HCC) had the fewest number of descriptions regarding palliative care. About 70–80% of HCC cases are associated with chronic hepatitis B or C infection, and there is often a long asymptomatic period [24]. Therefore, the HCC CPGs, rather than having descriptions about palliative care, had more descriptions about aggressive treatment such as hepatic resection and liver transplantation.

### Specific descriptions

In both the 2007 and 2015 surveys, the highest number of descriptions contained “helpful content”, specifically in regard to “pain” (Fig. 2). The descriptions about “pain” included substantial information about specific treatments such as nerve blocks and radiation therapy for bone metastases. In addition, references were made to specific cancer pain guidelines [22] in terms of treating “pain.” In a previous study, more than half of the CPGs discussed “natural history” and “non-pain symptoms” [13]. Our study investigated only cancer CPGs. Moderate to severe pain in cancer is common and affects 70–80% of patients with advanced disease, [30] so much has been written about “pain.” However, in this study, hardly any descriptions were found regarding psychological, social, or spiritual distress. The reason for this is probably because there are very few randomized trials or high quality observational studies that assess whether palliative care contributes to improved QOL in cancer patients [31, 32]. Descriptions of the above issues can be expected to increase as more high-quality evidence is acquired.

### CPGs in shared decision-making

Since the Cancer Control Act was enacted in 2007, CPGs describing terms related to palliative care have increased, and interest in palliative care appears to be increasing.

In palliative care, communication between the patient and the physician is important to understand patients’ intentions about where and how they wish to approach the end of life and whether these wishes can be carried out [33]. In helping patients and their families prepare for the end of life, CPGs, as decision-making support tools, must serve as a basis for shared decision-making among patients, their families, and healthcare providers. Cancer CPGs need be developed as support tools for palliative care communication between patients and physicians.

### Study limitations

This study was conducted using content analysis, with “line” used as a concrete index for the number of descriptions of “palliative care”. Although a consensus of definitions was reached among the co-investigators, there may have been some variation in the classifications. For example, for cancers with relatively gradual progression such as prostate cancer, there was often a direct mention of the “mortality rate.” Conversely, for cancers with rapid progression, such as pancreatic cancer, the use of the word “prognosis” to convey remaining time was noted. This may have resulted from different framing by investigators regarding the characteristics of cancer progression. In these instances, although mortality rate in “necrology” and prognosis in “natural history” may have similar meanings, they were counted as separate items. Thus, when a similar circumstance was expressed in two different ways, selected items may have been divided. Therefore, these variations were taken into consideration, and the results were carefully interpreted in relation to differences in the proportion of descriptions. In addition, if classification of “minimal content” and “helpful content” was difficult, it was regarded as “helpful content” This may account for the increased amount of “helpful content”. However, even when the above considerations were taken into account, a comparison between the two survey periods showed an increasing trend in the number of palliative care descriptions in the CPGs.

As a method of analysis, we used “content analysis,” which is an established qualitative analysis method. Therefore, our analysis is based on only the information included in CPGs. In AGREE II, which is a popular method for evaluating CPGs, only the content of description in CPGs is checked. Therefore, there is no target of analysis other than the information described above.

We considered palliative care as “care given to a patient when there is no response to curative treatment and life expectancy is less than one year” based on the content of cancer control programs, the situation in Japan in 2007, and descriptions in a previous study [13]. Although this definition does not correspond with the description “palliative care from the time when cancer is diagnosed” used in cancer control programs, we believe that indications in cancer diagnoses regarding how the perception of palliative care changed before and after the Cancer control Act have been enforced.

## Conclusions

After enactment of the Cancer Control Act, an increase was observed in the number but not in the proportion of palliative care descriptions in Japanese cancer CPGs. In the future, CPGs can be expected to play a role in helping cancer patients to incorporate palliative care smoothly by collaborating with individual clinical societies for various types of cancer and the Japanese Society for Palliative Medicine, which provides common ground.

## Abbreviations

BSC: Best Supportive care
CPGs: Clinical practice guidelines
HCC: Hepatocellular carcinoma
PEACE: Palliative care Emphasis program on symptom management and Assessment for Continuous medical Education
QOL: Quality of life
